# Selective Roles of Normal and Mutant Huntingtin in Neural Induction and Early Neurogenesis

**DOI:** 10.1371/journal.pone.0064368

**Published:** 2013-05-14

**Authors:** Giang D. Nguyen, Solen Gokhan, Aldrin E. Molero, Mark F. Mehler

**Affiliations:** 1 Roslyn and Leslie Goldstein Laboratory for Stem Cell Biology and Regenerative Medicine, Albert Einstein College of Medicine, Bronx, New York, United States of America; 2 Institute for Brain Disorders and Neural Regeneration, Albert Einstein College of Medicine, Bronx, New York, United States of America; 3 Department of Neurology, Albert Einstein College of Medicine, Bronx, New York, United States of America; 4 Department of Neuroscience, Albert Einstein College of Medicine, Bronx, New York, United States of America; 5 Department of Psychiatry and Behavioral Sciences, Albert Einstein College of Medicine, Bronx, New York, United States of America; 6 Rose F. Kennedy Center for Research on Intellectual and Developmental Disabilities, Albert Einstein College of Medicine, Bronx, New York, United States of America; 7 Einstein Cancer Center, Albert Einstein College of Medicine, Bronx, New York, United States of America; 8 Ruth L. and David S. Gottesman Institute for Stem Cell and Regenerative Medicine Research, Albert Einstein College of Medicine, Bronx, New York, United States of America; 9 Center for Epigenomics, Albert Einstein College of Medicine, Bronx, New York, United States of America; 10 Institute for Aging Research, Albert Einstein College of Medicine, Bronx, New York, United States of America; Instituto de Medicina Molecular, Portugal

## Abstract

Huntington's disease (HD) is a neurodegenerative disorder caused by abnormal polyglutamine expansion in the amino-terminal end of the huntingtin protein (Htt) and characterized by progressive striatal and cortical pathology. Previous reports have shown that Htt is essential for embryogenesis, and a recent study by our group revealed that the pathogenic form of Htt (mHtt) causes impairments in multiple stages of striatal development. In this study, we have examined whether HD-associated striatal developmental deficits are reflective of earlier maturational alterations occurring at the time of neurulation by assessing differential roles of Htt and mHtt during neural induction and early neurogenesis using an *in vitro* mouse embryonic stem cell (ESC) clonal assay system. We demonstrated that the loss of Htt in ESCs (KO ESCs) severely disrupts the specification of primitive and definitive neural stem cells (pNSCs, dNSCs, respectively) during the process of neural induction. In addition, clonally derived KO pNSCs and dNSCs displayed impaired proliferative potential, enhanced cell death and altered multi-lineage potential. Conversely, as observed in HD knock-in ESCs (Q111 ESCs), mHtt enhanced the number and size of pNSC clones, which exhibited enhanced proliferative potential and precocious neuronal differentiation. The transition from Q111 pNSCs to fibroblast growth factor 2 (FGF2)-responsive dNSCs was marked by potentiation in the number of dNSCs and altered proliferative potential. The multi-lineage potential of Q111 dNSCs was also enhanced with precocious neurogenesis and oligodendrocyte progenitor elaboration. The generation of Q111 epidermal growth factor (EGF)-responsive dNSCs was also compromised, whereas their multi-lineage potential was unaltered. These abnormalities in neural induction were associated with differential alterations in the expression profiles of *Notch*, *Hes1* and *Hes5*. These cumulative observations indicate that Htt is required for multiple stages of neural induction, whereas mHtt enhances this process and promotes precocious neurogenesis and oligodendrocyte progenitor cell elaboration.

## Introduction

HD is a neurodegenerative disorder caused by abnormal polyglutamine expansion in the amino-terminal end of huntingtin protein (Htt) and characterized by preferential striatal and cortical cellular dysfunction and death associated with late-onset neuropsychiatric and motor disabilities [1]. The molecular basis underlying the selective cellular vulnerability in HD and HD pathogenesis in general remains largely elusive. Htt is a large cytosolic protein of ∼340 kDa with ubiquitous cellular localization and adult functional pleiotropism, involving cellular processes ranging from transcriptional regulation to cell survival, whereas mutant Htt (mHtt) causes selective and progressive striatal and cortical neuronal dysfunction and subsequent cell death [2]–[6]. These observations suggest that Htt may mediate a distinct, selective and previously uncharacterized set of developmental functions, and the pathogenic mutation may therefore have the potential to deregulate these maturational processes and predispose to neurodegeneration. Identifying and characterizing this potential developmental diathesis may have important implications for defining an earlier HD pathogenic window, for explaining the occurrence of a protracted prodromal phase of the disease and for developing novel disease modifying therapies.

An increasing number of reports have begun to implicate Htt in seminal early neural developmental processes. For example, conditional deletion of the huntingtin gene (*htt*) in the whole brain (Hdh^flox/−^;Camk2a ^Cre/+^) from as early as embryonic day 14.5 (E14.5) causes widespread neurodegeneration that mimics several HD phenotypes [7]. Hypomorphic expression of Htt (Hdh^neoQ50^ homozygotes) leads to severe malformation of fore- and mid-brain regions, whereas the complete ablation of Htt results in embryonic lethality as early as E6.5 with a range of severe neural developmental defects, including impairments in the formation of the neural plate and absence of head-folds [8]–[12]. KO embryos also display a shortened primitive streak and absence of the embryonic organizer, which are essential for neural development [11]. These cumulative observations suggest that Htt has additional roles in earlier stages of neural induction and early neurogenesis. In line with these observations, a spectrum of neural developmental deficits have recently been demonstrated by our group in a HD knock-in mouse model (Q111) as early as E13.5, including impairments in striatal neural stem cell (NSC) maintenance, and NSC-mediated medium spiny neuron (MSN) specification and maturation [13]. Other reports of aberrant profiles of adult neurogenesis, including enhanced NSC proliferation, have also been documented in HD models and human pathological specimens [14]–[16]. Another recent study reported significantly reduced neuronal differentiation in HD knock-in embryonic stem cell (ESC)-derived NSCs with enhanced cell death [17]. In concert with these overall findings, there is increasing evidence of abnormalities of brain morphology, alterations in synaptic and neural plasticity, the presence of subtle neuropsychological impairments and other HD-associated manifestations occurring long before the advent of overt clinical deficits in HD patients and mouse models [18]–[27].

In the present study, we examined the roles of Htt during neural induction and early neurogenesis, and also assessed whether the HD pathogenic mutation (mHtt) may affect the integrity of these essential developmental processes. To accomplish this goal, we utilized an established ESC neural induction culture model that recapitulates the progressive stages of neural induction and early neurogenesis occurring *in vivo.*

## Results

### Htt is required for the specification, self-renewal and proliferation of LIF-responsive pNSCs, whereas mHtt enhances these processes and promotes precocious neurogenesis

**Figure 1:**
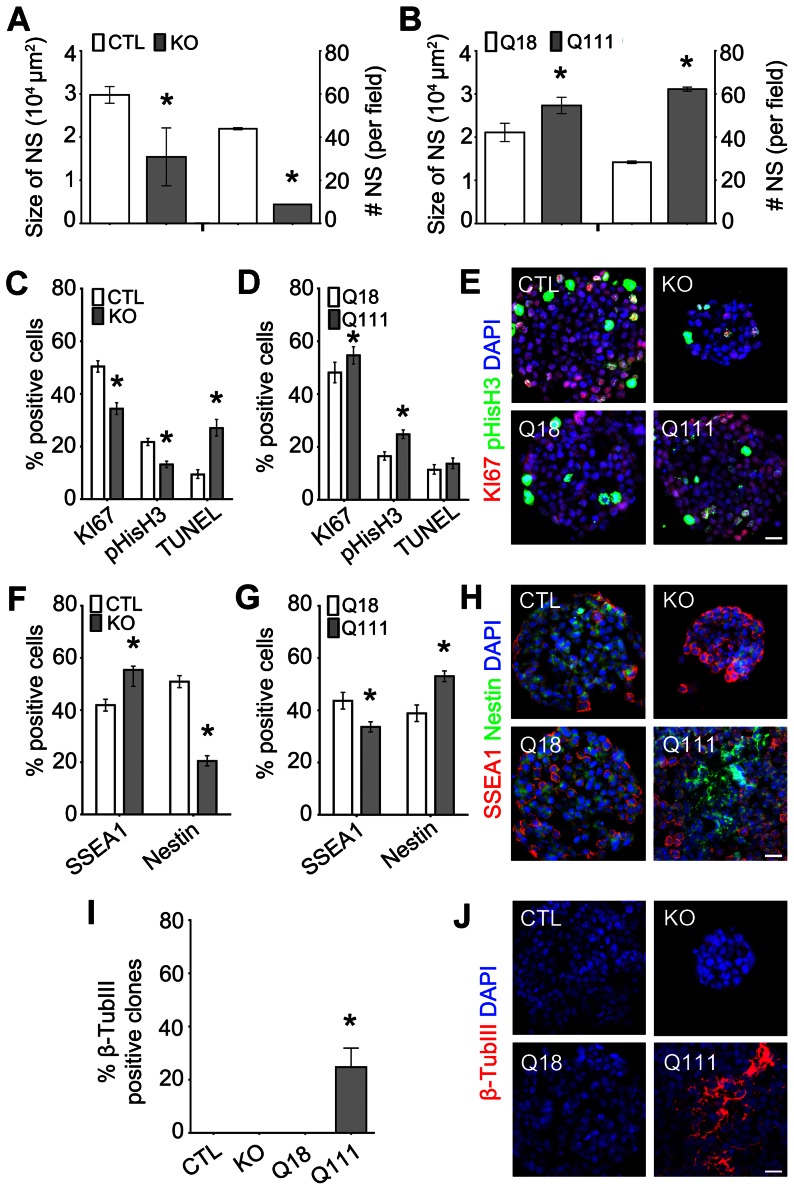
Htt is required for the elaboration of LIF-responsive pNSs, whereas mHtt differentially deregulates this process. (A, B) Quantification of the size and number of KO and Q111 pNSs as compared to CTL and Q18 pNSs, respectively. Error bars represent ± SEM; unless otherwise stated, *p-value<0.05. (C, D) Quantification of the percentage of positive cells for the proliferation markers, KI67 and pHisH3, and for the cell death marker, TUNEL, in KO pNSs as compared to CTL pNSs, and in Q111 pNSs as compared to Q18 pNSs, respectively. (E) Immunofluorescence micrographs of KI67 and pHisH3 immunoreactive cells in CTL, KO, Q18 and Q111 pNSs. (F, G) Quantification of the percentage of positive cells for the ESC marker, SSEA1, and the NSC marker, Nestin, in KO pNSs as compared to CTL pNSs, and in Q111 pNSs as compared to Q18 pNSs, respectively. (H) Immunofluorescence micrographs of SSEA1 and Nestin immunoreactive cells in CTL, KO, Q18 and Q111 pNSs. (I) Quantification of pNSs expressing the early neuronal marker, β-TubIII, from CTL, KO, Q18 and Q111 ESCs. (J) Immunofluorescence micrographs of β-TubIII immunoreactive in CTL, KO, Q18 and Q111 pNSs. Error bars represent ±95% CI; unless otherwise stated, *p-value<0.05. All scale bars = 25 μm.

Utilizing the ESC paradigm for neural induction, pNSCs can be identified with a colony-forming assay in the presence of leukemia inhibitory factor (LIF) in which they form clonally derived primitive neurospheres (pNSs) [28]. The pNSs express nestin and proneural genes while suppressing expression of the ESC marker, SSEA1 and alternate endodermal/mesodermal lineage genes [29]. To determine whether neural induction can normally occur in the absence of Htt, we compared ESC-derived pNSCs generated from Hdh^ex4/5^/Hdh^ex4/5^ ESCs, hereafter referred to as KO ESCs, and control ESCs, hereafter referred to as CTL ESCs [10]. Both the size and number of the KO pNSs were significantly smaller than the CTL pNSs (size: 1.5×10^4^ vs 3.0×10^4^ μm^2^ respectively, p = 0.0002; number: 9 vs 44 respectively, p-value<0.0001; Fig. 1A). In addition, KO pNSs were composed of significantly fewer KI67+ and phosphorylated histone H3 (pHisH3)+ cells, markers for dividing cells and G2/M-phase cells, respectively, as compared to CTL pNSs (34.4 vs 50.4%; 13.1% vs 22.7%, p-values<0.0001, respectively; Fig. 1C and E). Moreover, the percentage of dying cell, defined by TUNEL expression, was significantly higher in the KO pNSs (27.0 vs 9.4%, p-value<0.0001, respectively; Fig. 1C and Fig. S1A). Lineage analysis of the KO pNSs as compared to CTL pNSs also revealed persistent expression of SSEA1 and reduced expression of nestin, marker for ESCs and NSCs, respectively, thereby suggesting that the loss of Htt resulted in impairment of the ESC transition to pNSCs (SSEA1: 55.4 vs 41.9%; Nestin: 20.5 vs 50.9% for CTL and KO respectively, all p-values<0.0001; Fig. 1F and H).

However, when we compared Q111 ESCs, which has an expansion of 111 CAG repeats [30], with control Q18 ESCs, which express 18 CAG repeats, we observed an aberrant enhancement in the specification of pNSCs. Both the size and the number of Q111 pNSs were significantly increased as compared to Q18 pNSs (size: 2.7×10^4^ vs 2.1×10^4^ μm^2^; number: 62 vs 28, respectively, all p-values<0.0001, respectively; Fig. 1B). In addition, there was an increase in the percentage of proliferating cells that were KI67 and pHisH3 positive, while no differences were observed in the percentage of TUNEL positive dying cells in Q111 ESCs as compared to Q18 ESCs (Ki67: 54.7 vs 48.2%, p-value<0.0001; pHisH3: 24.8 vs 16.5%, p-value = 0.0127; TUNEL: 13.7 vs 11.4%, p-values = 0.0912, respectively; Fig. 1D and E; Fig. S1A). Moreover, compared to Q18 pNSs, there was a significant increase in the percentage of nestin+ cells and a concomitant reduction in SSEA1+ cells in Q111 pNSs (SSEA1: 33.7 vs 43.7%; nestin: 53.9 vs 38.1%, respectively, all p-values<0.0001; Fig. 1G and H). It has previously been shown that only about 1% of all pNSCs express β-TubIII; however, about 20% of all Q111 pNS displayed expression of β-TubIII, which indicates the presence of precocious neurogenesis in the presence of mHtt (Fig. 1I and J). These overall observations suggest that Htt is required for the incipient program of neural induction as well as for the self-renewal, proliferation and survival capacity of LIF-responsive pNSCs. Furthermore, the mutation in Htt enhances ESC-derived neural induction and leads to precocious neuronal lineage specification.

### Htt is required for the specification, self-renewal and proliferation of FGF2- and EGF-responsive dNSCs, whereas mHtt differentially alters these processes and further promotes precocious neurogenesis in FGF2-responsive dNSCs

**Figure 2:**
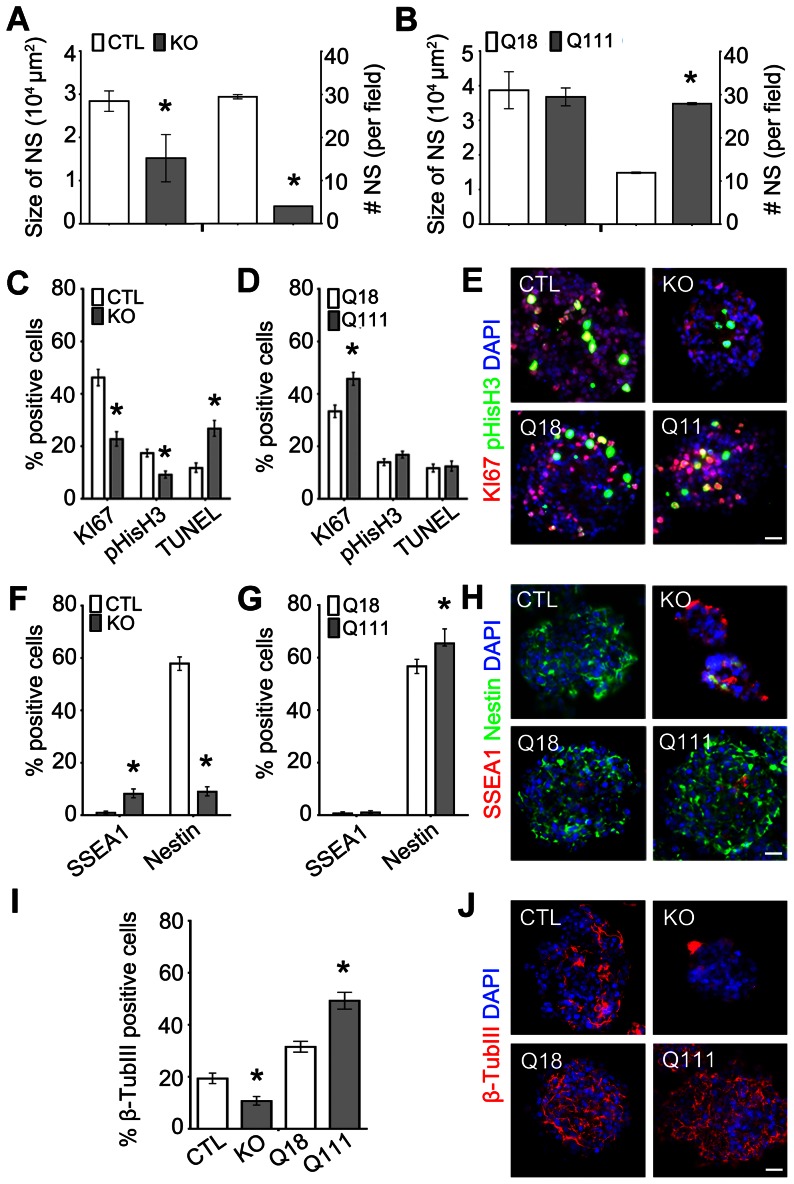
Htt is required for the elaboration of FGF2-responsive dNSs, whereas mHtt differentially deregulates this process. (A, B) Quantification of the size and number of KO and Q111 pNSs as compared to CTL and Q18 FGF2-responsive dNSs, respectively. Error bars represent ± SEM; unless otherwise stated, *p-value<0.05. (C, D) Quantification of the percentage of positive cells for proliferation markers, KI67 and pHisH3, and for the cell death marker, TUNEL, in KO FGF2-responsive dNSs as compared to CTL FGF2-responsive dNSs, and in Q111 FGF2-responsive dNSs as compared to Q18 FGF2-responsive dNSs, respectively. (E) Immunofluorescence micrographs of KI67 and pHisH3 immunoreactive cells in CTL, KO, Q18 and Q111 FGF2-responsive dNSs. (F, G) Quantification of the percentage of positive cells for the ESC marker, SSEA1, and the NSC marker, Nestin, in KO FGF2-responsive dNSs as compared to CTL FGF2-responsive dNSs, and in Q111 FGF2-responsive dNSs as compared to Q18 FGF2-responsive dNSs, respectively. (H) Immunofluorescence micrographs of SSEA1- and Nestin-immunoreactive cells in CTL, KO, Q18 and Q111 FGF2-responsive dNSs. (I) Quantification of the percentage of total positive cells for the early neuronal marker, β-TubIII, in CTL, KO, Q18 and Q111 FGF2-responsive dNSs. (J) Immunofluorescence micrographs of β-TubIII-immunoreactive cells in CTL, KO, Q18 and Q111 FGF2-responsive dNSs. Error bars represent ±95% CI; unless otherwise stated, *p-value<0.05. All scale bars = 25 μm.

We next examined the role of Htt in the program of neural specification of dNSCs and assessed whether the presence of mHtt alters this developmental program. Individual LIF-responsive pNSCs were dissociated and re-propagated in the presence of FGF2 to generate FGF2-responsive dNSCs [29], [31]. These dNSCs are equivalent to their *in vivo* counterparts that exist from E8.5 through adulthood. Analogous to the developmental profiles observed with KO pNSs, both the size and number of the KO FGF2-responsive dNSs were significantly decreased as compared to CTL FGF2-responsive dNSs (size: 3.0×10^4^ vs 5.7×10^4^ μm^2^; number: 4 vs 29, respectively, all p-values<0.0001, respectively; Fig. 2A). Consistently, KO FGF2-responsive dNSs were composed of significantly fewer numbers of KI67+ and pHisH3+ cells than CTL FGF2-responsive dNSs, whereas the percentage of TUNEL+ cells remained significantly higher (Ki67: 22.7 vs 46.2%; 9.1 vs 17.3%; TUNEL: 26.7 vs 11.7%, respectively, all p-values<0.0001; Fig. 2C and E; Fig. S1B). Immunofluorescence lineage analysis of KO FGF2-responsive dNSs also revealed a significantly lower percentage of nestin+ NSCs and β-TubIII+ neuronal precursors with a higher percentage of SSEA1+ ESCs as compared to CTL FGF2-responsive dNSs (SSEA1: 8.1 vs 1.0%; Nestin: 8.9 vs 57.9%; β-TubIII: 10.7 vs 19.3%, respectively, all p-values<0.0001; Fig. 2F, H and I). In contrast to the findings with the KO ECSs, the presence of mHtt resulted in significantly higher numbers of Q111 FGF2-responsive dNSs than those of Q18 FGF2-responsive dNSs even though there was no difference in their respective sizes (number: 27.9 vs 11.9, respectively, p-value<0.0001; size: 3.7×10^4^ vs 3.9×10^4^ μm^2^, respectively, p-value = 0.0521; Fig. 2B). In addition, as compared to Q18 FGF2-responsive dNSs, there was an increase in the percentage of KI67+ and pHisH3+cells in Q111 FGF2-responsive dNSs, whereas the percentage of TUNEL+ cells was unchanged (Ki67: 45.7 vs 33.3%, p-value<0.0001; pHisH3: 16.8 vs 14.0%, p = 0.0019; TUNEL: 12.3 vs 11.7%, p-value = 0.5760, respectively; Fig. 2D-F and Fig. S1B). Further lineage analysis revealed that Q111 FGF2-responsive dNSs also displayed significantly higher proportions of nestin+ NSCs and β-TubIII+ neuronal precursors as compared to the Q18 FGF2-responsive dNSs (nestin: 65.4 vs 56.7%; β-TubIII: 49.3 vs 31.6%, relatively, all p-values<0.0001; Fig. 2G-J). The increase in the percentage of β-TubIII+ cells suggests an enhanced specification of committed neuronal progenitors. These cumulative observations suggest that Htt is required for the transition of LIF-responsive pNSCs to FGF2-responsive dNSCs and for the promotion of self-renewal, proliferation and neuronal lineage fate of FGF2-responsive dNSCs. Conversely, mHtt enhanced the transition from LIF-responsive pNSCs to FGF2-responsive dNSCs with alterations in proliferative potential and precocious neurogenesis.

FGF2-responsive dNSCs are the direct precursors of EGF-responsive dNSCs [31]. To further investigate the role of Htt and the effects of mHtt in the specification of EGF-responsive dNSCS from FGF2-responsive dNSCs, we dissociated and re-propagated FGF2-responsive dNSs in EGF to generate EGF-responsive dNSs. Both the number and size of the KO EGF-responsive dNSs were significantly decreased as compared with CTL EGF-responsive dNSs (size: 0.1×10^4^ vs 3.5×10^4^ μm^2^; number: 0 vs 9, respectively, all p-values<0.0001; Fig. S1C). Upon differentiation after 7 days *in vitro* (DIV), few irregularly shaped EGF-responsive dNSs were formed from KO ESCs and these clones failed to differentiate into neurons and glia (Fig. S1E). In contrast, the size of Q111 EGF-responsive dNSs was comparable to Q18 EGF-responsive dNSs, whereas the number of Q111 EGF-responsive dNSs was significantly decreased as compared to those of Q18 EGF-responsive dNSs (2.0×10^4^ vs 1.8×10^4^ μm^2^, p-values = 0.5550; 1 vs 2; p-values<0.0001, respectively, Fig. S1D). Nonetheless, the elaboration of β-TubIII+ neuronal species and GFAP+ astrocytes in Q111 EGF-responsive dNSs were comparable to those in the Q18 EGF-responsive dNSs under differentiating conditions. (Fig. S1E). These observations indicate that Htt is required for the developmental transition of FGF2-responsive dNSCs to EGF-responsive dNSCs and the subsequent differentiation into neurons and glia, whereas mHtt selectivel*y* impairs the specification of EGF-responsive dNSCs but does not alter their neural lineage potential.

### Htt is required for the expression of ectodermal and neural genes, and the repression of genes specifying alternate endodermal cell fate in LIF-responsive pNSCs, whereas mHtt selectively enhances ectodermal and neuronal gene expression

**Figure 3:**
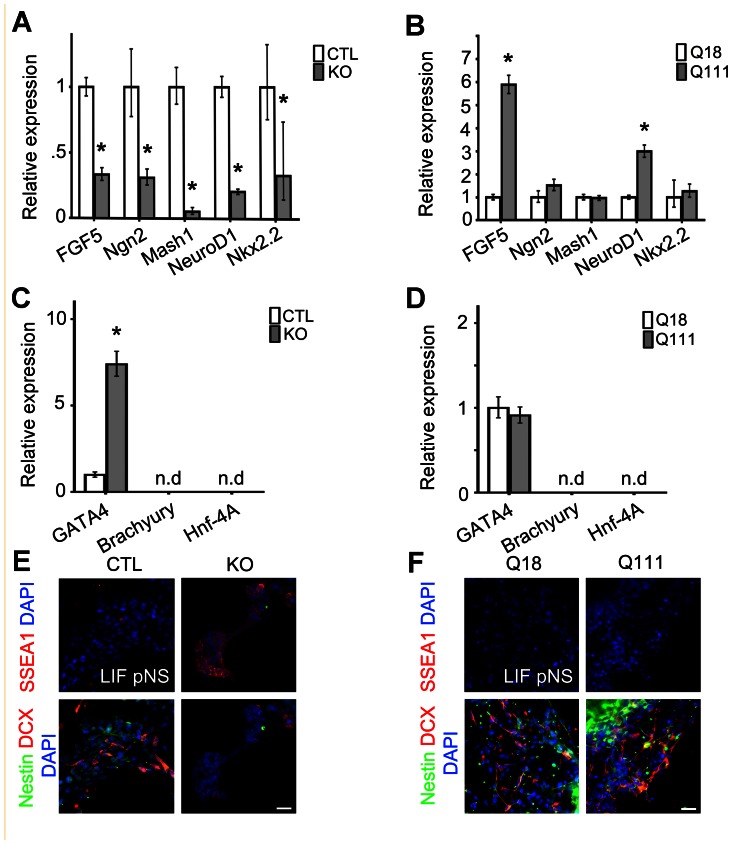
Htt is required for the maintenance of lineage potential of pNSCs, whereas mHtt promotes neurogenesis. (A, B) QPCR expression analysis of proneural genes in KO pNSs as compared to CTL pNSs, and in Q111 pNSs as compared to Q18 pNSs, respectively. (C, D) QPCR expression analysis of the endodermal gene, *GATA4*, and the mesodermal genes, *Brachyury* and *Hnf-4A*, in KO pNSs as compared to CTL pNSs, and in Q111 pNSs as compared to Q18 pNSs, respectively. (E, F) pNSs were cultured under differentiating conditions for 7DIV and analyzed by immunofluorescence microscopy for SSEA1, Nestin and Doublecortin (DCX), which are markers for ESCs, NSCs and neuronal precursor species respectively. Error bars represent ±95% CI; unless otherwise stated, *p-value<0.05. All scale bars = 25 μm.

To investigate the roles of Htt and mHtt in mediating lineage potential during the process of early neural induction, we assessed expression levels of genes involved in promoting neural and non-neural lineage decisions from pNSCs, and further examined NSC maintenance and lineage potential under differentiating conditions. KO pNSCs exhibited significant downregulation in the expression level of the primitive ectoderm gene, *FGF5* (0.333, p-value<0.001), the proneural genes, *Ngn2* and *Mash1* (0.31 and 0.055, respectively; p<0.001), the neurogenic gene, *NeuroD1* (0.207, p-value<0.001) and the early patterning and gliogenic gene, *Nkx2.2* (0.329, p-value = 0.012), as compared to CTL pNSCs (Fig. 3A). Additionally, contrary to previous studies reporting that wild type pNSCs do not express any endodermal or mesodermal genes [28], the expression of the endodermal gene, *GATA4,* was significantly increased in KO pNSs as compared to CTL pNSs (7.387, p-value<0.001), thereby suggesting Htt modulates the repression of endodermal lineages during neural induction in ESCs (Fig. 3C). Furthermore, immunofluorescence lineage analysis after 7DIV under differentiating conditions revealed that in contrast to CTL pNSCs, KO pNSCs retained SSEA1 expression and failed to express nestin and the neuronal precursor marker, doublecortin (DCX) (Fig. 3E). Conversely, gene expression analysis in Q111 pNSs revealed a significant upregulation in the expression levels of the primitive ectodermal gene, *FGF5* (5.888, p-value<0.001) and the neurogenic gene, *NeuroD1* (2.989, p-value<0.001), as compared to Q18 pNSs (Fig. 3B). Additionally, there were no discernible differences in the expression levels of genes involved in specifying the alternate endodermal lineage (GATA4, 0.875, p-value = 0.071) when comparing Q111 and Q18 pNSs (Fig. 3D). Further, after 7DIV under differentiating conditions, the expression of SSEA1 was completely repressed in Q111 pNSs, whereas there were no significant differences in the specification of nestin+ NSCs and DCX+ neuronal precursor species between Q111 and Q18 pNSs (Fig. 3F). These experimental findings suggest that Htt is required for promoting ectodermal, and later neural lineage fates and for preventing the elaboration of selective alternate lineage (e.g., endodermal) fates, whereas mHtt enhances the expression of ectodermal and neurogenic genes in pNSCs that may have resulted in precocious neurogenesis. However, increases in the proportion of β-TubIII-expressing cells between Q18 and Q111 ESCs without corresponding differences in the profiles of DCX-expressing cells suggest that mHtt is promoting precocious neuronal specification but not progressive neuronal maturation.

### Htt is required for the expression of ectodermal and neural genes, and the repression of genes specifying alternate mesodermal cell fates in FGF2-responsive dNSCs, whereas mHtt selectively alters ectodermal and neural gene expression and promotes precocious oligodendrocyte specification

**Figure 4:**
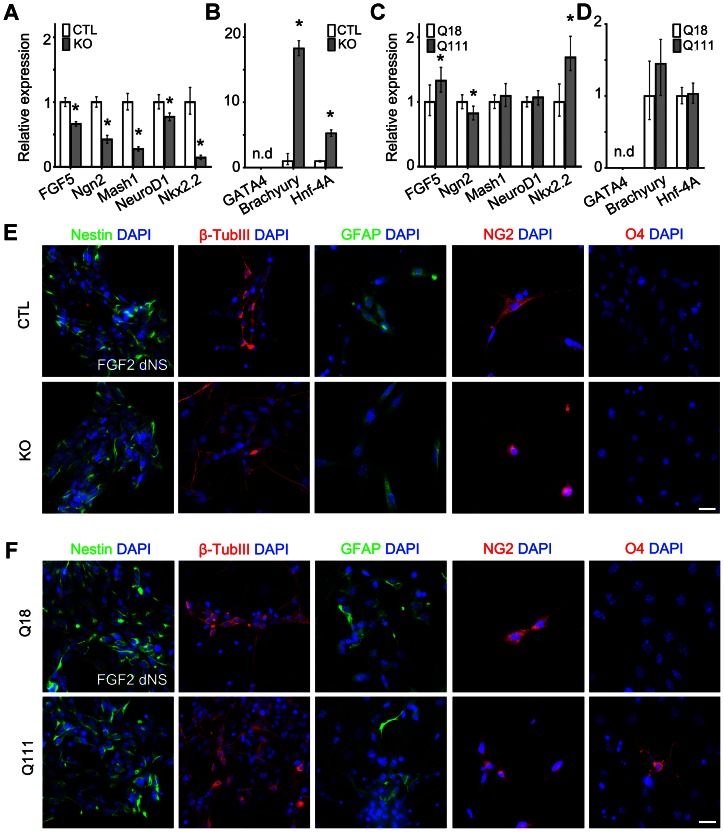
Htt is required for the maintenance of lineage potential in dNSCs, whereas mHtt selectively deregulates this process. (A, C) QPCR expression analysis of proneural genes in KO FGF2-responsive dNSs as compared to CTL FGF2-responsive dNSs, and in Q111 FGF2-responsive dNSs as compared to Q18 FGF2-responsive dNSs, respectively. (B, D) QPCR expression analysis of the endodermal gene, *GATA4*, and the mesodermal genes, *Brachyury* and *Hnf-4A*, in KO FGF2-responsive dNSs as compared to CTL FGF2-responsive dNSs, and in Q111 FGF2-responsive dNSs as compared to Q18 FGF2-responsive dNSs, respectively. (E, F) FGF2-responsive dNSs were cultured under differentiating conditions for 7DIV and analyzed by immunofluorescence microscopy for assessment of the expression profiles of Nestin, β-TubIII, GFAP, NG2 and O4, which are markers for NSCs, neurons, astrocytes, oligodendrocyte precursors and oligodendrocyte progenitors, respectively. Error bars represent ±95% CI; unless otherwise stated, *p-value<0.05. All scale bars = 25 μm.

To further investigate the roles of Htt and mHtt in mediating neural and non-neural lineage potential in FGF2-responsive dNSCs, we again examined the profiles of developmental gene expression and the propensity of FGF2-responsive dNSCs for displaying multi-neural lineage potential under differentiating conditions. As compared to the CTL FGF2-responsive dNSs, gene expression analysis of KO FGF2-responsive dNSs revealed downregulation of ectodermal and pro-neural genes (FGF5: RQ = 0.663, p-value<0.001; Ngn2: RQ = 0.425, p-value<0.001; Mash1: RQ = 0.278, p-value<0.001; NeuroD1: RQ = 0.773, p-value = 0.001; Nkx2.2: RQ = 0.145, p-value<0.001) in concert with significant upregulation in the expression of mesodermal genes, *Brachyury* (RQ = 18.267, p-value<0.001) and *Hnf-4A* (RQ = 5.268, p-value<0.001) (Fig. 4A and B). Interestingly, after 7DIV under differentiating conditions, the small number of irregularly shaped KO FGF2-responsive dNSs gave rise to similar profiles of nestin+ NSCs. However, compared to CTL, KO FGF2-responsive dNSs exhibited decreased proportions of β-TubIII+ neurons (22.8% vs 16.4%, respectively, p-value<0.0001) and GFAP+ astrocytes (0.6% vs 0.1%, respectively, p-value <0.0001) but a similar proportion of NG2+ oligodendrocyte (OL) precursors (1.4% vs 1.2%, respectively) (Fig. 4E). In addition, as compared to CTL, KO FGF2-responsive dNSs contained a smaller proportion of unipotent neuronal clones (100% vs 33.3%, respectively, p-value<0.0001) and bipotent neuronal-astrocyte clones (34.8% vs 4.2%, respectively, p-value = 0.0093). On the other hand, gene expression analysis of Q111 FGF2-responsive dNSs revealed significant upregulation in the expression levels of the ectodermal gene, *FGF5* (RQ = 1.329, p-value = 0.039) and the early patterning and gliogenic gene, *Nkx2.2* (RQ = 1.885, p-value = 0.019), as compared to Q18 FGF2-responsive dNSs (Fig. 4C). Consistently, there were no statistical differences in the expression profiles of selective endodermal and mesodermal genes when comparing Q111 to Q18 FGF2-responsive dNSs (GATA4: not detected; Brachyury: RQ = 1.448, p = 0.083; Hnf-4A: RQ = 1.216, p-value = 0.755; Fig. 4D). After 7DIV under differentiating conditions, as compared to Q18, Q111 FGF2-responsive dNSs exhibited an increased complement of β-TubIII+ neurons (19.6% vs 23.5%, respectively, p-value<0.0001), GFAP+ astrocytes (0.2% vs 0.6%, respectively, p-value<0.0001) and NG2+ oligodendrocyte (OL) precursors (1.1% vs 1.2%, respectively). In addition, as compared to Q18, Q111 FGF2-responsive dNSs exhibited a modest increase in the proportion of O4+ OL progenitor species (0.07% vs 0.3%, respectively, p-value<0.0001), potentially due to the increased expression of *Nkx2.2* as observed previously (Fig. 4F). There was no significant difference in the proportion of unipotent and bipotent clones between Q18 and Q111 FGF2-responsive dNSs. Interestingly, as compared to Q18 FGF2-responsive dNSs, Q111 dNSs exhibited a large proportion of multipotent clones (0% vs. 35.3%, respectively). These experimental findings indicate that Htt is required for the expression of ectodermal and pro-neural genes and for the repression of genes associated with mesodermal fate during the transition from LIF-responsive pNSCs to FGF2-responsive dNSCs. This is associated with corresponding reductions in the elaboration of neuronal and glia lineages, as well as significant but selective reductions in unipotent neuronal and bipotent neuronal-astrocyte clonal potential. By contrast, mHtt enhances ectodermal and selective pro-neural gene expression, and enhances the elaboration of neuronal and glial lineages with a selective increase in multilineage potential.

### Htt is associated with Notch signaling pathways during the specification and maintenance of pNSCs and dNSCs, whereas mHtt differentially deregulates this developmental signaling cascade

**Figure 5:**
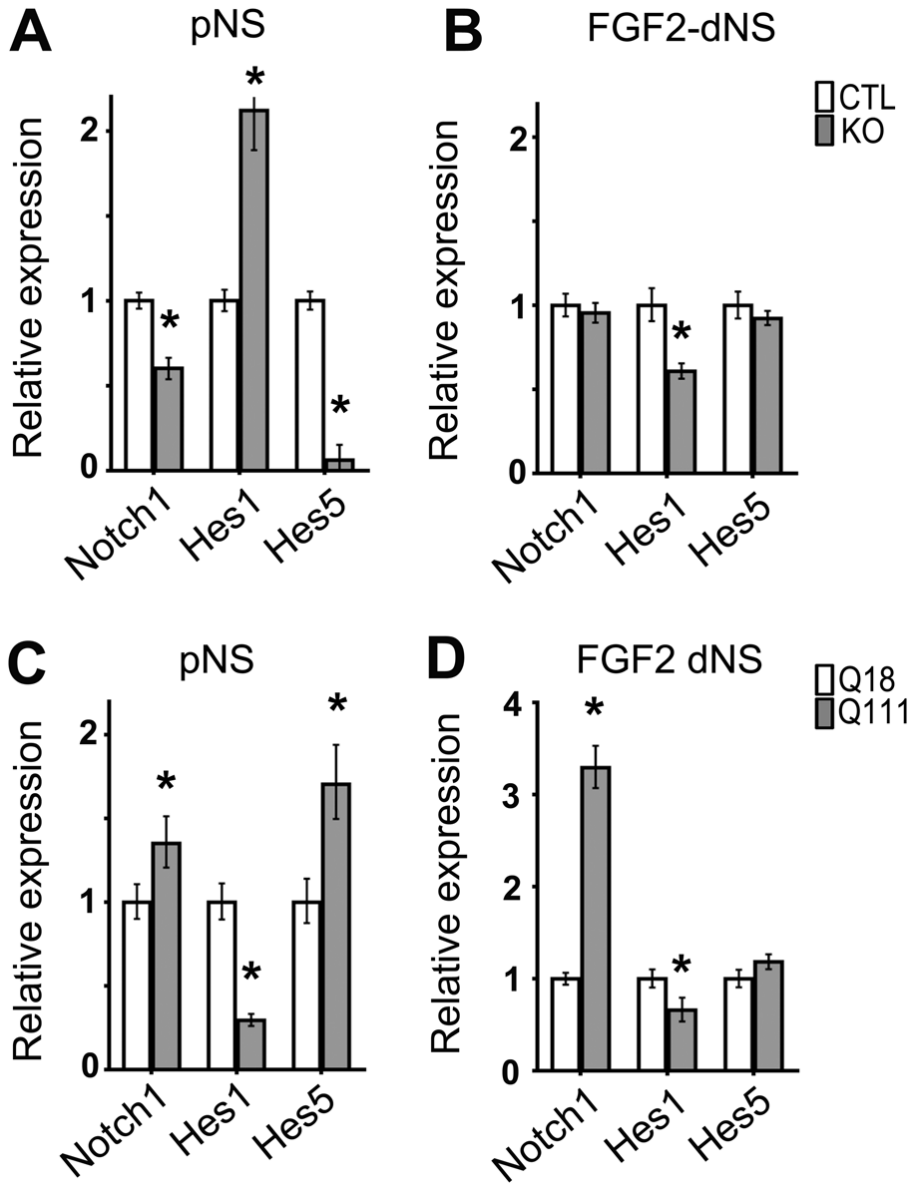
Htt is associated with Notch signaling pathways, whereas mHtt differentially deregulates this signaling cascade. (A, B) QPCR expression analysis of *Notch*, *Hes1* and *Hes5* in KO as compared to CTL pNSs and dNSs. (C, D) QPCR expression analysis of *Notch*, *Hes1* and *Hes5* in Q111 as compared to Q18 pNSs and dNSs. Error bars represent ±95% CI; unless otherwise stated, *p-value<0.05.

Notch signaling pathways play pivotal roles in cell fate diversification during development. In particular, the Notch/Hes pathway is essential for the transition of pNSCs to dNSCs and NSC proliferation and maintenance [32], [33]. To determine whether Htt is required for the integrity of Notch signaling during the process of neural induction, we analyzed the expression profiles of *Notch*, *Hes1* and *Hes5* in both KO pNSCs and dNSCs. Gene expression analysis showed that both *Notch* (RQ = 0.603, p-value<0.001) and *Hes5* (RQ = 0.062, p-value<0.001) expression were significantly downregulated in KO pNSCs, whereas the expression of *Hes1* (RQ = 2.119, p-value<0.001) was significantly upregulated as compared to control CTL pNSCs (Fig. 5A). However, the expression levels of *Notch* (RQ = 0.995, p-value = 0.233) and *Hes5* (RQ = 0.923, p-value = 0.097) in KO FGF2-responsive dNSCs were comparable to CTL FGF2-responsive dNSCs, whereas the expression of *Hes1* (RQ = 0.609, p-value<0.001) was significantly downregulated (Fig. 5B). By contrast, for Q111 pNSCs, *Notch* (RQ = 1.352, p-value<0.001) and *Hes5* (RQ = 1.705, p-value<0.001) expression levels were significantly upregulated, whereas the level of expression of *Hes1* (RQ = 0.296, p-value<0.001) was significantly downregulated as compared to Q18 pNSCs (Fig. 5C). Although the expression level of *Hes1* (RQ = 0.662, p-value = 0.012) remained significantly downregulated as Q111 LIF-responsive pNSCs transitioned to FGF2-responsive dNSCs, *Notch* expression levels became significantly upregulated (RQ = 3.291, p-value<0.001; Fig. 5D). These observations suggest that Htt modulates Notch signaling pathways during the specification and maintenance of pNSCs and dNSCs, and mHtt differentially disrupts *Notch/Hes1/Hes5* signaling during both pNSC and dNSC developmental stages.

## Discussion

In this study, we employed a specialized ESC clonal culture paradigm to characterize the entire program of neural induction and early neurogenesis [28], [29], and demonstrated the essential roles of Htt in the program of neural induction, progressive specification of neural progenitor cell types and the subsequent elaboration of neural lineage species. Our study also revealed that the HD pathogenic mutation aberrantly enhanced ESC-derived neural fate specification, resulting in precocious neurogenesis of pNSCs, and enhanced the elaboration of neuronal and glial lineages from dNSCs.

The development of the central nervous system (CNS) begins with the early program of neural induction within the anterior region of the epiblast. It has been shown that neural fate specification in the pre-gastrula epiblast exists as a ‘default’ state and FGF signaling from the organizer antagonizes the inhibitory effects of bone morphogenetic proteins (BMPs) on anterior neural fate [34]. However, following gastrulation, the organizer further ‘induces’ the elaboration and patterning of neural tissue by antagonizing other neural inhibitory signals, such as Nodal and Wnt [35]. Although Htt has previously been shown to be essential for neural development at the time of gastrulation, the roles of Htt for early neural induction have not been adequately explored, in part, due to the early lethality of the KO embryo [11]. Furthermore, the presence of severe mesodermal impairments in KO embryos prevents definitive assessment of the direct roles of Htt in neural development, as these mesodermal structures play critical inductive roles for neural development [35]. The *in vitro* clonal ESC neural induction model recapitulates the *in vivo* program of neural induction that follows the “default” pathway in the absence of confounding extrinsic factors [28], [29]. Thus this experimental paradigm provides an important alternative approach that circumvents many of the aforementioned experimental limitations. However, control conditions for the KO and Q111 ESC lines differed in several lineage parameters. These observations are likely due to the fact that the appropriate controls differed by the presence of mouse (R1 ESC) and human (Q18) 5′ sequences within the huntingtin gene, which did not exhibit complete homology. The utilization of separate controls for the KO and Q111 ES cell conditions was necessary because the mutant huntingtin ESC line was constructed with the humanized expansion repeat sequence.

The early stage of LIF-responsive pNSC induction *in vitro,* however, is a particularly vulnerable developmental phase due to the enhanced sensitivity of apoptosis signaling pathways to caspase-mediated cell death [28], [29]. Htt has been shown to display primary anti-apoptotic functions that are mediated, in part, through direct inhibition of activation of caspase 3 and 9, and therefore the absence of Htt may enhance the cellular vulnerability of the KO pNSCs [5]. The presence of LIF has also been demonstrated to have important pro-survival roles in pNSCs, and Htt is known to interact with Grb2 and RasGAP, two adapter molecules of the LIF receptor [36]. Thus, the absence of Htt may disrupt the LIF receptor-mediated pro-survival pathway and further impair the survival of the KO pNSCs. Interestingly, these adapter molecules are also part of the FGF receptor-signaling pathway that is essential for the specification and proliferation of pNSCs and their transition to dNSCs [28], [37].

The Notch pathway is an active signaling cascade regulating the early program of neural induction. Our study has shown that the absence of Htt disrupted expression of *Notch* as well as *Hes5*, an essential Notch effector, in KO pNSCs. By contrast, the ablation of *Notch* (Notch^−/−^) in mouse embryos has been demonstrated to only reduce *Hes5* expression, but does not disrupt the generation of pNSCs [33]. This strongly indicates that the requirement of Htt for the specification of pNSCs is independent from its putative role in modulating Notch signaling pathways. Alternatively, high Hes1 in KO pNSCs can suppress proliferation as it has been shown that high Hes1 levels in neural progenitors can repress cyclin D1 and result in G1 phase retardation [38]. Additionally, and consistent with our observations, high Hes1 expression levels have also been shown to promote preferential mesodermal differentiation over neural differentiation, possibly via repression of Notch signaling [39], [40].

Interestingly, Notch^−/−^ embryonic brains as well as Notch^−/−^ ESCs are severely impaired in the generation of FGF2- and EGF-responsive dNSCs [33]. Similarly, knockdown of *RBP-Jκ*, a downstream mediator of Notch signaling, has also been shown to deplete early dNSCs [32]. Thus, the reduced *Notch*/*Hes5* expression we observed in KO pNSCs may have resulted in impairment in their transition to dNSCs. However, some KO pNSCs were capable of undergoing early neural developmental transition to form FGF2-responsive dNSs, which also displayed comparable levels of expression of *Notch* and *Hes5* as the controls. As appropriate *Notch* expression levels are important to direct ESC differentiation to neural lineages [41], the apparent normal levels of expression of *Notch* and *Hes5* in KO FGF2-responsive dNSs suggest that Htt may not play a role in the regulation of Notch signaling in dNSCs, and that alterations in neuronal and glial lineage elaboration may be due, in part, to additional non-redundant developmental signaling pathways. Thus far, Htt has not been shown to have any direct interaction with components of the Notch signaling cascade, with the exception of a single study reporting an indirect functional association between Huntingtin interacting protein 1 (HIP-1) and deltex-dependent Notch signaling in *Drosophila* that plays a role in neurogenesis [42]. Additional studies are required to elucidate the mechanisms underlying this regulatory function.

Htt may also play an important role at the intersection of neural and non-neural fate decisions during the incipient program of neural induction as both KO pNSCs and dNSCs displayed preferential increases in mesodermal and endodermal gene expression over pro-neural gene expression. These specialized roles of Htt in cell fate decisions may be orchestrated by modulating the functions of the neuron-restrictive silencing factor/RE1-silencing transcription factor (NRSF/REST) by normally sequestering it within the cytoplasm [43]. REST is a transcriptional and epigenetic regulator of both neural and non-neural cell fate specification programs [43]. It has previously been demonstrated that overexpression of REST in ESCs can promote early differentiation of ESC-derived embryoid bodies to primitive endoderm and also disrupt specification of the epiblast [44]. Further studies are required to show whether the loss of Htt may enhance aberrant accumulation of REST in the nucleus and contribute to the preferential acquisition of endodermal over ectodermal fates during the program of neural induction.

On the other hand, in Q111 pNSCs the presence of mHtt enhanced *Notch* and *Hes5* expression levels. Interestingly, enhanced FGF receptor signaling in dNSC can also potentiate Notch signaling and enhance neurogenesis [45], [46]. Constitutive Notch activation (NotchIC) has been shown to not only upregulate Hes5 expression levels but also more importantly to enhance the generation of dNSCs, which is consistent with our observations in Q111 dNSCs [33]. These dNSCs then progressively become Notch/Hes5-dependent and undergo asymmetric cell division to modulate the balance between the maintenance of NSC populations and neural lineage commitment [47], [48]. Thus, the sustained increase in the expression of *Notch* in Q111 dNSCs may differentially enhance asymmetric cell divisions resulting in premature specification of committed neural progenitors, which is consistent with our observation of enhanced generation of neuronal and glial lineages [47], [49]–[51]. Furthermore, this may also lead to the premature depletion of Q111 FGF2-responsive dNSCs, and thus to deficits in the generation of Q111 EGF-responsive dNSCs. Conversely, *Hes1* expression levels were significantly downregulated in Q111 pNSCs and dNSCs and may have contributed to the preferential expression of both neuroectodermal and neurogenic genes, to enhanced proliferative capacity and to precocious neurogenesis. Indeed, low *Hes1* expression in ESCs has been shown to preferentially enhance neural differentiation, whereas the complete ablation of Hes1 further promoted premature neurogenesis [40], [49]. High *Hes1* expression has also been reported to have suppressive effects on the maturation of NG2+/O4- OL precursors, which is consistent with the enhanced elaboration of O4+ OL progenitors in the low *Hes1-*expressing Q111 dNS culture condition [51]. The latter observation may have important implications for defining the mechanistic underpinnings of previous findings of increased oligodendrocyte density reported in the caudate nucleus in HD patients [52]. Remarkably, Notch signaling, particularly with respect to Notch1/3, has been shown to play pivotal roles in the developmental stage-specific regulation of neural progenitors in the ventricular zone that contribute to striatal development [53]. These findings suggest that mHtt alters Notch signaling cascades during neural induction, and these and related molecular pathways may have important implications for explaining the regional striatal developmental deficits previously reported by our group in the HD knock-in Q111 mouse model [13].

Our findings of significant alterations in proliferative potential, self-renewal as well as neural and non-neural lineage potential in Q111 pNSCs and dNSCs have important implications for HD. First, these cellular alterations may result in impairments in neural lineage specification in neurogenic zones that, in part, is consistent with several reports of enhanced self-renewal and precocious neurogenesis in the subventricular zone (SVZ) of R6/2 and Hdh-Q150 KI HD mouse models [14], [54]. Second, highly proliferative Q111 pNSCs undergo enhanced DNA replication and are therefore at increased risk for accelerated DNA damage and repair responses, which have been shown to promote mutational instability of CAG repeats and potentially contribute to the pathogenesis of HD [55], [56]. Putative DNA instability may persist in mutant pNSCs and subsequently in their progeny, thereby promoting the propagation of developmental mutation length-mediated cellular and functional impairments into adult life. These pathogenic possibilities are consistent with several reports of increased DNA instability and CAG expansion mosaicism in the brains of HD patients and mouse models [55], [57].

A recent study by Conforti and colleagues reported that the loss of Htt and the presence of mHtt (NS-Hdh^ex4/5^ and NS-Hdh^Q111/7^, respectively) did not disrupt the *in vitro* derivation of ESC-derived NSCs or impair their self-renewal and proliferative properties. In addition, the Hdh^Q111/7^ NSCs were shown to display reduced neurogenesis and increased cell death [17]. The differences observed between these findings and those of the present study may stem from the use of alternate experimental protocols. Importantly, the current study extends our previous published observations in Q111 mice that mHtt deregulates cell cycle parameters of NSCs and results in aberrant expansion of intermediate progenitors in the absence of increased cell death [13]. Furthermore, the previous work from our group [13] and the current findings strongly suggest that HD-associated abnormalities in adult life (reviewed in [58]) may stem from early and cumulative neurodevelopmental impairments, and may therefore support the notion that HD represents a primary neurodevelopmental disorder in addition to a neurodegenerative disease [59]. Equally important is the concept that seminal impairments occurring during early stages of the neural developmental program can potentially lead to multiple foci of regional cellular vulnerabilities along the entire neuraxis, observations increasingly shown to be associated with HD and other neurodegenerative disease phenotypes [26], [27], [60]–[63].

It is imperative to corroborate the observations in this study with other *in vivo* HD models to better refine our understanding of the potential contributions of the HD pathogenic mutation and of differing numbers of pathogenic expansion repeats during incipient stages of embryonic and neural development. Moreover, it is also important to define key molecular impairments occurring along the continuum of developmental and adult stages in affected individuals and in robust HD animal models by identifying potentially unique developmental protein partners of Htt, such as regulators of transcriptional, epigenetic and additional diverse cellular processes. These essential initiatives will open up new possibilities for innovative and efficacious diagnostic, therapeutic and preventative strategies for HD.

## Materials and Methods

### Embryonic Stem Cell Culture Paradigms

The KO, Q18 and Q111 ESCs (Hdh^ex4/5^/Hdh^ex4/5^, Hdh-Q18 and Hdh-Q111, respectively), were previously generated and graciously supplied by MacDonald et al. [10], [30]. The R1 ESC line from ATCC was used as the control (CTL). ESCs were maintained on mouse embryonic fibroblast (MEF) feeder layers that had previously been inactivated with Mitomycin C (Sigma, M4287). Prior to use for specific experimental protocols, ESCs were plated and maintained on 0.1% gelatin-coated tissue culture plates in ES cell media consisting of knockout Dulbecco's minimal essential medium (Invitrogen, DMEM, 10313) supplemented with 1000 U/ml of leukemia inhibitory factor (LIF/ESGRO; Chemicon, ESG1106), 10% ES-qualified FBS (ATCC, SCRR-30-2020), 1X MEM nonessential amino acids (from 100x stock, Invitrogen 11140), 1X L-glutamine and antibiotics (from 100x stock, Invitrogen 10378-016), and 0.1 mM 2-mercaptoethanol (Sigma, M7522).

### ESC-derived Primitive and Definitive NSC Assays

These assays were carried out as previously described [28]. Briefly, all culture conditions were carried out in serum-free media that consisted of DMEM/F-12 (Invitrogen, 11330) supplemented with 25 ug/ml insulin (Sigma, I6634), 100ug/ml transferrin (Sigma, T1147), 20 nM progesterone (Sigma, P7556), 60 μM putrescine (Sigma, P5780), 30 nM sodium selenium (Sigma, S5261), 1x L-glutamine and antibiotics (100x stock, Invitrogen 10378-016), 5 mM Hepes (Sigma, H3375) and 3mM NaHCO_3_ (Sigma, S5761). ESCs were plated as single-cell suspensions at densities < = 10 cells/μl on uncoated culture plates using the above media formulation supplemented with LIF (1000U/ml; Chemicon, ESG1106) for 7 days *in vitro* (DIV) to form LIF-responsive pNSs. The pNSs were dissociated into single cells by trypsin/0.04% EDTA (Invitrogen, 25300-054) and re-plated in the same media supplemented with 10ng/ml FGF2 (BD; 354060) and 2 μg/ml Heparin (Sigma-Aldrich) for another 7 DIV to form FGF2-responsive dNSs. The FGF2-responsive dNSs were further dissociated and re-plated in the presence of 20ng/ml EGF for an additional 7 DIV to form EGF-responsive dNSs. For differentiation paradigms, individual clonal spheres were plated onto Matrigel-coated plates in same media supplemented with 1% FBS.

### Immunofluorescence Analysis

NSs were collected by centrifugation at 300 rpm for 5 minutes, washed once in PBS and fixed in 4% PFA for 20 minutes at room temperature. NSs were then collected in 20% sucrose until they became totally submerged and then frozen in M-1 Embedding Matrix (Thermo) for cryo-sectioning. Immunofluorescence analysis was carried out as previously described [13], [64] (See Table S1 for the list of utilized antibodies). TUNEL analysis was performed according to the manufacturer's protocols (Roche, 11684795910). BrdU analysis was carried out as previously described [13].

### Quantitative Real-Time PCR (QPCR)

Harvesting of RNA from samples was carried out using TRI reagent® (Molecular Research Center Inc, Cincinnati, OH, USA) according to manufacturer's protocol. The quantification of total RNA concentration was determined using the Qubit® RNA assay kit and Qubit® 2.0 Fluorometer (Invitrogen). Single strand cDNA synthesis was performed using the High Capacity RNA Reverse Transcription Kit® (Applied Biosystems, 4368814) following the manufacturer's recommendations. TaqMan primers were purchased from PE Applied Biosystems and SYBR Green probes were generated using the Invitrogen service (See Table S1). We utilized either TaqMan Universal PCR Master Mix® or SYBR Green Master Mix and ran samples in triplicate in the Model 7000 Real Time PCR system® (Applied Biosystems, CA, USA). The housekeeping gene employed was hypoxanthine guanine phosphoribosyl transferase 1 (*HPRT1*). Data collection and quality assessment were performed utilizing the 7000 SDS 1.1 RQ Software (Applied Biosystems, CA, USA). The analysis was accomplished with the 2(-Delta-Delta C(T)) relative quantification method with the Relative Expression Software Tool (REST) developed by Corbett Research [65–66]. Gene expression levels were reported using the relative RQ values with ±95% Confidence Interval (CI).

### Statistical Analysis

Statistical comparisons were evaluated according to the type of data analyzed: proportions were compared with Chi-square test or Fisher's Test. The means of samples were analyzed with either Mann-Whitney U test or *t-*test. Statistically significant differences between samples were considered using a probability of at least <0.05.

## Supporting Information

Figure S1 **TUNEL assays and the roles of Htt and mHtt in the elaboration of EGF-responsive dNSs.** (A, B) Immunofluorescence micrographs of TUNEL-immunoreactive cells contained within CTL, KO, Q18 and Q111 pNSs and FGF2-responsive dNSs. (C, D) Quantification of the size and number of CTL, KO, Q18 and Q111 EGF-responsive dNSs. (E) EGF-responsive dNSs were cultured under differentiating conditions for 7DIV and analyzed by immunofluorescence microscopy for the expression profiles of the neuronal and astrocyte markers, β-TubIII and GFAP, in CTL, KO, Q18 and Q111 clones. Error bars represent ±95% CI; unless otherwise stated, *p-value<0.05. All scale bars = 25 μm.
(TIF)

Table S1 **List of antibodies, TaqMan probes and SYBR Green probes utilized in the study.** All antibodies are listed with manufacturers' names, catalogue numbers, as well as concentration used. All TaqMan probes are listed with catalogue numbers from Applied Biosystems. All SYBR Green probes are listed with forward and reverse sequences.
(DOCX)
