# Supplementary figures and images for: Selective Roles of Normal and Mutant Huntingtin in Neural Induction and Early Neurogenesis
Source: PLoS One. 2013 May 14;8(5):e64368. doi: 10.1371/journal.pone.0064368 (PMC3653864; doi:10.1371/journal.pone.0064368)

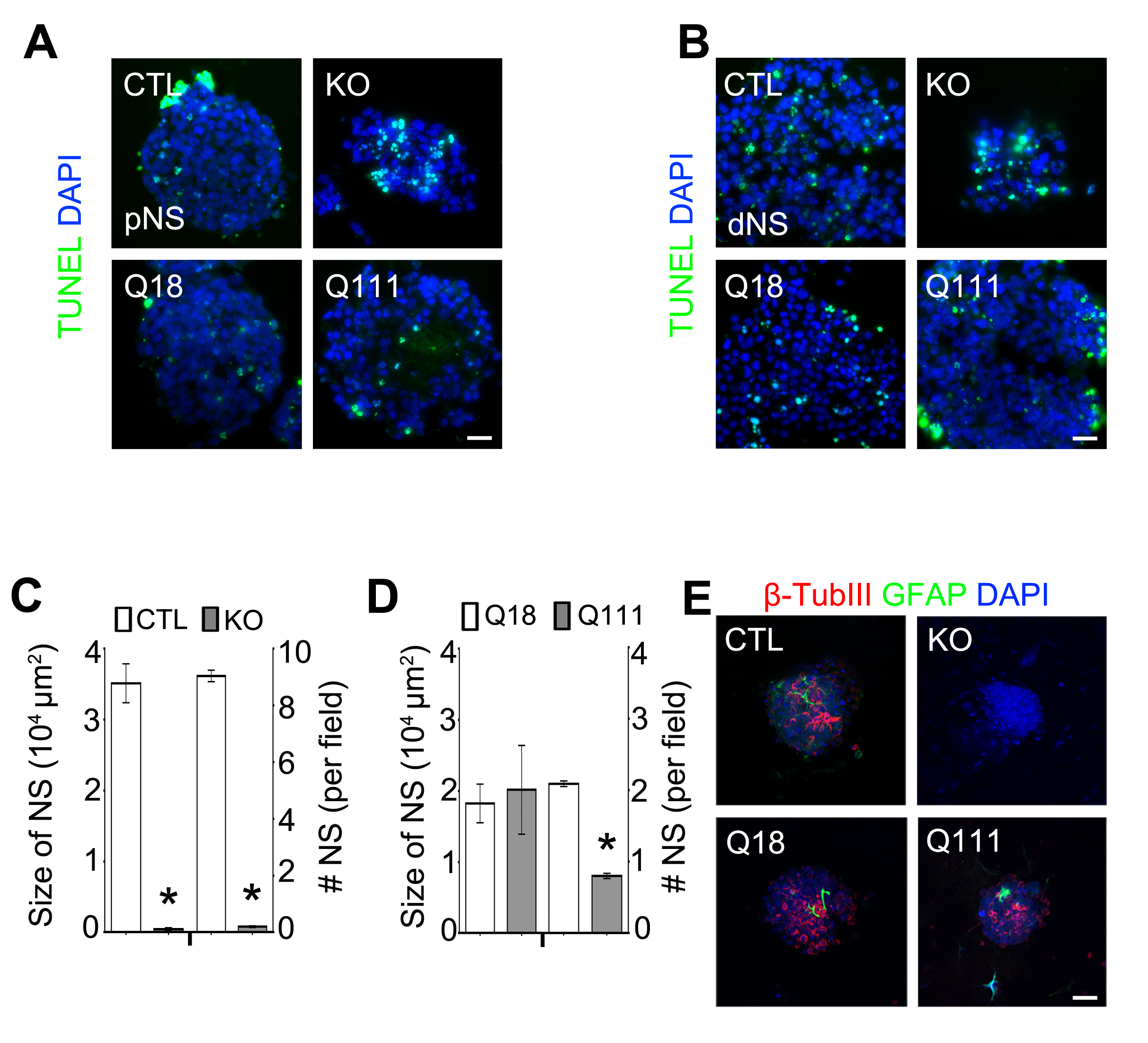

Supplement: Figure S1 — TUNEL assays and the roles of Htt and mHtt in the elaboration of EGF-responsive dNSs. (A, B) Immunofluorescence micrographs of TUNEL-immunoreactive cells contained within CTL, KO, Q18 and Q111 pNSs and FGF2-responsive dNSs. (C, D) Quantification of the size and number of CTL, KO, Q18 and Q111 EGF-responsive dNSs. (E) EGF-responsive dNSs were cultured under differentiating conditions for 7DIV and analyzed by immunofluorescence microscopy for the expression profiles of the neuronal and astrocyte markers, β-TubIII and GFAP, in CTL, KO, Q18 and Q111 clones. Error bars represent ±95% CI; unless otherwise stated, *p-value<0.05. All scale bars = 25 μm. (TIF) [file pone.0064368.s001.tif]
