# Supplementary material for: Selective Roles of Normal and Mutant Huntingtin in Neural Induction and Early Neurogenesis
Source: PLoS One. 2013 May 14;8(5):e64368. doi: 10.1371/journal.pone.0064368 (PMC3653864; doi:10.1371/journal.pone.0064368)
Supplement: Table S1 — List of antibodies, TaqMan probes and SYBR Green probes utilized in the study. All antibodies are listed with manufacturers' names, catalogue numbers, as well as concentration used. All TaqMan probes are listed with catalogue numbers from Applied Biosystems. All SYBR Green probes are listed with forward and reverse sequences. (DOCX) [file pone.0064368.s002.docx]

**Table S1**

| **Antibody** | **Isotype/Concentration** | **Manufacturer / Cat. #** |  |
| --- | --- | --- | --- |
| SSEA-1 | IgM / 1:50 | Hybridoma Bank, MC-480 |  |
| Nestin | IgG1 / 1:200 | B&D, 556309 |  |
| βIII-Tubulin | IgG2B / 1:800 | Sigma, T8660 |  |
| Double-cortin (DCX) | Goat / 1:400 | Santa Cruz, sc-8066 |  |
| GFAP | IgG1 / 1:400 | Sigma, G3893 |  |
| NG2 | Rabbit / 1:100 | Millipore, AB5320 |  |
| O4 | IgM / 1:350 | Sigma, O7139 |  |
| KI67 | IgG1 / 1:100 | Novo Castra, NCL-L-KI67-MM1 |  |
| pHisH3 | Rabbit / 1:100 | Millipore, 06-570 |  |
| **TaqMan Probes** | **Applied Biosystems catalogue number** | | |
| HPRT1 | Mm00446968_m1 | | |
| Notch1 | Mm00435245_m1 | | |
| Hes1 | Mm00468601_m1 | | |
| Hes5 | Mm00439311_g1 | | |
| FGF5 | Mm00438918_m1 | | |
| **SYBR Green Probes** | **Forward/Reverse Sequences** | | |
| HPRT1 | CAGTCCCAGCGTCGTGATTA / GAATAAACACTTTTTCCAAATCCTCG | | |
| Ngn2 | CCGCGTAGGATGTTCGTCAAA / GACATCGGGGTCAGGGTCG | | |
| Mash1 | CTACGACCCTCTTAGCCCAG / TGCCATCCTGCTTCCAAAGTC | | |
| NeuroD1 | ACCTTTTAACAACAGGAAGTGGA / CTCATCTGTCCAGCTTGGGG | | |
| Nkx2.2 | ACCTGGCCAGCCTCATCCGT / TGAAATGCTTTCTCCGCCCGGG | | |
| GATA4 | ACTGGAGCTGGCCAGGACTG / CTGCTACACACCCAGGCGCA | | |
| Brachyury | ACGGCAGGAGGATGTTCCCGG / GCGGTGGTTGTCAGCCGTCA | | |
| Hnf-4A | GCTGCAGAGCATCACCTGGCA / TGGCAGACCCTCCGAGAAGCA | | |
